# Feasibility and acceptability of strategies aiming to increase smoking cessation rates among people experiencing homelessness: A scoping review

**DOI:** 10.18332/tpc/236153

**Published:** 2026-10-01

**Authors:** Bertrand Redonnet, Giulia Muriago, Solenne Bonneterre, Aurélia Roversi, Alexandre R. Gomajee, Camilla Somma, Maria Melchior

**Affiliations:** 1 Sorbonne Université, INSERM, Sorbonne Public Health Institute, ESSMA (Épidémiologie Sociale, Santé Mentale et Addictions) Paris France; 2 Università degli Studi di Verona (UNIVR) Verona Italy

**Keywords:** smoking cessation, scoping review, people experiencing homelessness

## Abstract

**Introduction:**

People experiencing homelessness (PEH) have high smoking rates, limited access to smoking cessation support, and face specific challenges in quitting tobacco. To date, optimal strategies to reduce smoking rates in this marginalized population are not clearly identified. This study aims to identify the feasibility and acceptability of smoking cessation interventions evaluated among PEH.

**Methods:**

We assessed evidence regarding the feasibility and acceptability of interventions promoting smoking cessation among persons aged >18 years, experiencing homelessness, and who smoke tobacco. We included primary quantitative, qualitative, and mixed-methods studies informing on PEH’s and professionals’ attending to their needs, experiences, and perspectives. We searched the scientific literature (PubMed, Web of Science) published up to 1 July 2026.

**Results:**

A total of 55 articles and six intervention categories were identified: 1) counseling/motivational interviewing (MI); 2) contingency management (CM); 3) text messaging-based interventions (TMI); 4) e-cigarettes; 5) collaborative professional models; and 6) smoking cessation policies. Most interventions aimed at increasing participants’ motivation to quit smoking. Counseling/MI and CM were the most frequently tested programs, showing limited to moderate results. TMI had limited results with regard to smoking relapse. All tested interventions were deemed acceptable by participants. Patient-centered and harm reduction approaches were particularly appreciated.

**Conclusions:**

This scoping review shows that counseling/MI and CM, which appear to have limited to moderate effectiveness in terms of smoking cessation support among PEH, are well accepted; however, their feasibility is questionable – especially CM. Other forms of smoking cessation support show lower levels of effectiveness. There is a need for further research on effective and acceptable ways of promoting smoking cessation among marginalized groups such as PEH.

## Introduction

Smoking remains a public health issue worldwide, with negative impacts on the health of individuals and societal well-being. Efforts to reduce smoking prevalence have shown success in recent decades across high-income countries^1^, but marginalized populations still exhibit disproportionately high rates of tobacco use^2,3^.

Among these groups, people experiencing homelessness (PEH) have high smoking rates^4^, estimated between 57% and 82%^5^. This represents an intersection of social, economic, and health factors that contribute to persistent inequality in smoking prevalence and persistence. Not only is tobacco smoking a major cause of premature death and illness, but it is also a contributor to disadvantage and health inequalities^6,7^.

Among PEH, tobacco smoking is responsible for a high prevalence of chronic obstructive pulmonary disease, heart problems, and illnesses of the respiratory system^8,9^. Tobacco-attributable mortality in this group is considerably higher than in the general population, and smoking is the leading cause of death among PEH aged 50 years^4^.

Despite high smoking rates, PEH are less likely to benefit from evidence-based smoking cessation interventions. Homeless smokers generally express the wish to quit smoking^10,11^, but compared to the general population they are much less likely to be successful in doing so^12,13^. This calls for *ad hoc* approaches, both individual and structural, for PEH. A systematic review^5^ and a Cochrane review^14^, both published in 2020, showed that counseling/motivational interviewing (MI) – an interview technique, as well as contingency management (CM) – financial incentives encouraging smoking cessation, show limited to moderate effectiveness in promoting smoking cessation among PEH. While Soar et al.^5^ presented barriers and facilitating factors for each type of smoking cessation intervention examined, feasibility and acceptability have not previously been described.

The present study aims to identify the feasibility and acceptability of smoking cessation interventions evaluated among PEH. Because of wide heterogeneity in study designs, methods, and findings, and to integrate qualitative as well as quantitative research findings, we conducted a scoping review^15^. Prior to discussing feasibility and acceptability, we briefly present evidence of effectiveness.

## Methods

To map the existing scientific literature on interventions promoting tobacco cessation among PEH, we followed methodological guidelines relative to the conduct of scoping reviews^16,17^. The study protocol was registered on the Open Science Framework (Registration DOI: 10.17605/OSF.IO/CKNUV). Subsequently, the following changes to the pre-registered protocol were made: 1) we do not report tobacco use prevalence rates; and 2) we only focus on intervention studies. Additionally, database searches were updated since the date indicated in the protocol. PICO (Population, Intervention, Comparison, and Outcomes) elements were determined as follows.

### Population

People aged >18 years, experiencing homelessness, smoking tobacco, who were offered a smoking cessation intervention. Homelessness encompasses multiple situations: sleeping rough, temporary accommodation in shelters, hostels, squats, camps, ‘hidden’ accommodation with friends or family, and we used each study’s own definition. We excluded research focused on formerly homeless individuals.

### Intervention

We studied the feasibility and acceptability of all interventions, divided into the following categories: 1) counseling/motivational interviewing (MI); 2) contingency management (CM); 3) text messaging interventions (TMI); 4) e-cigarettes for smoking cessation; 5) collaborative professional models; and 6) smoking cessation policies. Our scoping review included: 1) intervention studies; 2) randomized controlled trials (RCTs); 3) qualitative or mixed-methods studies; and 4) cross-sectional and longitudinal surveys reporting on the acceptability of an intervention. We excluded systematic reviews, study protocols with no published results, commentaries, editorials, and opinion papers.

### Comparison

Interventions are compared to standard or usual care, which may involve the provision of pharmacotherapy and psychosocial support. Some surveys or qualitative studies with outcomes of interest may have no comparator.

### Outcome

At individual level, we considered: 1) smoking abstinence during the intervention or at follow-up; 2) reduction in the number of cigarettes smoked; 3) smoking cessation attempts; and 4) motivation to quit smoking. At the collective level, we considered: 1) number of persons in smoking cessation treatment; 2) referral/access to smoking cessation and harm reduction services; 3) nicotine replacement therapy (NRT) uptake; and 4) use of e-cigarettes as a substitute for smoking. We also included qualitative studies documenting key themes related to PEH’s experiences and perceptions of smoking cessation, or practices of professionals in charge of this marginalized group. Secondary data analyses and cross-sectional studies were excluded if they did not report on smoking cessation outcomes of specific interventions.

### Search strategy

PubMed and Web of Science were searched up to 1 July 2026, with a combination of keywords relative to homelessness and smoking cessation interventions. Our search was not limited in terms of language. All retrieved records were referenced in Zotero.

Additionally, we manually searched references cited in the 2020 Cochrane review on smoking and homelessness^14^. We also searched ClinicalTrials.gov and the Cochrane Library to identify additional registered trials. Other eligible sources were located using backward searches of references.

The query strings used to search each database were: (homeless* OR housing instabilit* OR unhoused* OR unsheltered* OR unsettl*) AND (smokeless tobacco OR smoking cessation OR smoking reduction OR reducing smoking OR tobacco reduction OR tobacco cessation OR tobacco OR smok*)(in abstract) for Pubmed and (homeless* OR housing instabilit* OR unhoused* OR unsheltered* OR unsettl*) AND (smokeless tobacco OR smoking cessation OR smoking reduction OR reducing smoking OR tobacco reduction OR tobacco cessation OR tobacco OR smok*)(in abstract) for Web of Science.

### Screening

Abstracts were independently screened by two reviewers (BR and GM) blinded to one another, using Rayyan. In case of inter-rater differences, we attempted reconciliation, and if needed, a third reviewer intervened (MM).

### Data extraction

The data extraction tool was developed jointly by the two reviewers (BR and GM) and applied to all selected articles. Data extracted included: first author, year, study design, participants, age, sample size, aim of the study, details of the intervention, the category of intervention, and main findings relevant to effectiveness, feasibility, and acceptability of the studied smoking cessation intervention.

## Results

Our search yielded 2141 articles, of which 1181 were removed as duplicates with Rayyan. We screened the title and abstract of 960 records, resulting in 210 records kept for full-text screening. Coincidentally, all included publications were in English.

We excluded 156 full-text articles for the following reasons: not an intervention, wrong publication type, publication not available (eight studies we were unable to obtain the full text for, even after making a request to the authors, were excluded). One additional article was identified in a reference list.

Overall, this scoping review included 55 articles (Figure 1), based on 32 different studies. Supplementary file Appendix 1 lists complete citations for included articles: 46 articles were quantitative, 8 were qualitative, and one used mixed methods; 45 articles reported data from the USA, 6 from the UK, 3 from Australia, and one from Ireland; 29 articles examined intervention outcomes, 14 examined feasibility and acceptability, and 12 examined participant attitudes and perceptions; 49 articles concerned PEH only, 4 concerned professionals only, and 2 concerned both PEH and professionals. Three articles specifically concerned young PEH, and three concerned PEH who are US veterans.

**Figure 1:**
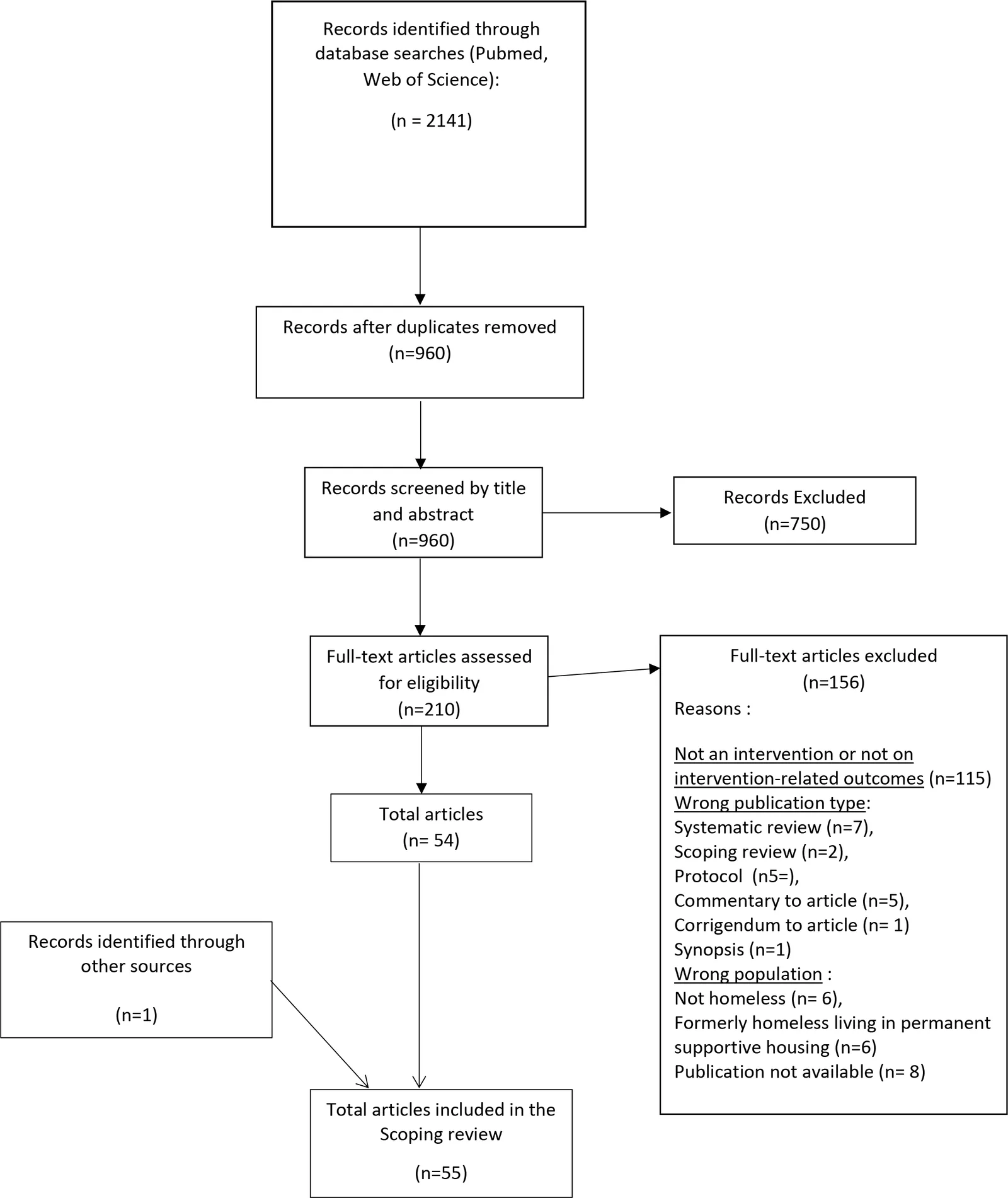
Flowchart showing selection of articles relative to smoking cessation interventions among people experiencing homelessness

Included studies were classified by type of intervention: counseling/MI, CM, TMI, e-cigarettes, collaborative smoking cessation promotion models, and smoking cessation policies. Each category includes both primary intervention studies and secondary analyses if they relate to smoking cessation outcomes, as well as information about feasibility and acceptability.

### Counseling/MI

Counseling/MI interventions covered multiple approaches and techniques aiming to support long-term behavior change through guidance, emotional support, motivation, and strategies to manage other substance use^18^ aiming to increase smoking cessation or reduction, as measured by breath carbon monoxide or the number of cigarettes per day. This category included 21 articles reporting on 9 different studies, the largest category in this review.

The 21 articles in this category are of the following types: nine primary studies (3 based on the RCT *Power to Quit* (PTQ) that recruited 430 participants across 8 homeless shelters and transitional housing units in Minnesota, USA; one report from the PTQ2 RCT and 6 pre-post-non-randomized intervention studies), six secondary analyses, three qualitative studies, a report on the implementation of an RCT, and a survey on its acceptability.

All studies were US-based, except one from Australia, and targeted adults experiencing homelessness. Most evaluated MI, a client-centered, directive technique aiming to resolve ambivalence towards smoking cessation and increase motivation for behavior change^19^. Three studies used individual in-person MI, one telephone-based MI, and two group MI. The PTQ RCT tested the effects of adding MI to NRT, while one study tested MI in combination with NRT and Varenicline. Another study evaluated the feasibility of 6 weekly sessions of MI conducted over the telephone.

Studies examining group MI assessed the impact of the intervention on commitment to smoking cessation and some integrated MI with cognitive-behavioral therapy (CBT). Three studies tested non-MI counseling approaches: one combined counseling following American Cancer Society guidelines with NRT, another trained shelter staff to provide brief smoking cessation counseling, and a qualitative study offered brief counseling and NRT at large events; 19 out of the 21 studies in this category provided participants with some form of NRT/pharmacotherapy, nicotine patches mostly for eight or sometimes twelve weeks. Varenicline was administered for 4 weeks and provided in combination with NRT in an Australian study conducted among homeless men attending a health clinic. Treatment adherence was low, in contrast with the results of a single-arm pilot study of group MI which reported low adherence to NRT, but high adherence to varenicline and bupropion. Supplementary file Table 1 lists the characteristics of studies based on counseling/MI.

#### Evidence for smoking cessation outcomes

Overall, counseling/MI showed limited or moderate effectiveness in reducing smoking rates among PEH. The PTQ RCT showed no statistically significant differences in NRT adherence nor in 7 day point-prevalence of smoking abstinence at 26 weeks. However, more intensive counseling protocols, such as those implemented in a pilot RCT for PTQ and in a single-arm pilot study tested among users of a shelter and a transitional residential treatment program in New York, NY, USA, led to promising 7 day point-prevalence of smoking abstinence rates. In the PTQ2 study, an intensive tobacco and alcohol behavioral intervention favored participants’ smoking outcomes compared to standard care (SC), but the difference was not statistically significant. Another study showed that group MI favored NRT adherence compared to NRT alone, but again these differences were not statistically significant.

The effects of MI on participants’ smoking cessation vary with other characteristics such as baseline motivation to quit^20^, the level of nicotine dependence and psychiatric comorbidities (e.g. depression, substance abuse). Emotional factors can also play a role: positive affect predicts a lower number of cigarettes per day (CPD), with strongest effects observed early in the quit attempt process, as shown in a study of 57 PEH recruited from a Dallas, TX, USA, shelter. However, long-term impact on craving remains unclear. In a secondary analysis of the PTQ RCT, negative affect and stress hindered smoking cessation, in part cued by exposure to smoking in the shelter vicinity. Another secondary analysis of the PTQ trial found that smokers with social relations with quitters are more likely to cease smoking successfully.

#### Feasibility

Key strategies enhancing the feasibility of smoking cessation trials with an MI component include flexible scheduling, multiple contact methods, and recruitment from shelters and transitional housing units. In secondary analyses of the PTQ RCT, staff play a crucial role in coordinating visits and in providing space for smoking cessation activities, while community mobilizers help foster trust and participation. Long-term follow-up strategies, including reminders and incentives (e.g. gift cards and transport passes), may improve participants’ engagement.

A qualitative study among PTQ2 participants found that barriers to MI intervention adherence include characteristics of the shelter environment (e.g. lack of privacy) and external factors such as scheduling conflicts and participants’ limited financial resources. Participants also described feeling pressure to smoke and drink alcohol in and around shelters.

Regarding non-MI counseling, implementation in transitional shelters in San Diego, CA, USA, along with smoke-free policies, improved intervention consistency and relevance. A community-based study in San Francisco, CA, USA, demonstrated it is possible to reach a large number of PEH in large-scale community events. Finally, a pre-post-intervention study examining a telephone-based health promotion intervention in Australia showed cost-effectiveness.

#### Acceptability

Counseling/MI interventions were generally considered acceptable by participants, especially those with a higher willingness to quit smoking. However, in a pilot study in New York, NY, USA, acceptability was lower among persons with lower motivation to quit, underscoring the importance of the preparation for the implementation of smoking cessation programs.

Counseling appears most beneficial when accompanied by NRT, as in the PTQ2 RCT. Initial reluctance to counseling gave way to appreciation of the emotional support received and increased confidence in the ability to manage smoking behaviors.

In the PTQ RCT, highest satisfaction levels were reported by African-American participants, likely due to culturally concordant staff. In the San Francisco area, CA, USA, participants in smoking cessation events reported positive experiences following brief counseling and appreciated being introduced to new cessation resources, such as bupropion. Finally, strong engagement was linked to the development of trust, emotional support, and positive staff relationships, identified as key factors to favor smoking cessation and alcohol abstinence in PTQ2.

### Contingency management (CM)

Ten articles based on seven different studies examined contingency management: one RCT, two pilot studies, one pilot RCT, one mixed-design study, one comparative effectiveness trial, two secondary analyses of RCTs, and two qualitative studies, including one based on an RCT. Some studies integrated CM in a broader strategy that also included MI. Financial incentives used to support sustained smoking cessation included gift cards, mailed checks, or random prizes. In seven out of ten articles, participants were also provided with NRT: nicotine patches for varying durations (five, eight or twelve weeks) or bupropion for up to twelve weeks. Supplementary file Table 2 lists the characteristics of studies on CM evaluated among PEH.

#### Evidence for smoking cessation outcomes

Overall, interventions implementing CM showed low to moderate effectiveness for smoking cessation. An RCT conducted in the northeastern USA among 70 PEH showed that participants receiving CM had significantly longer smoking abstinence and initially significantly higher CO-negative levels. However, 6 months after the intervention, smoking abstinence rates were similar in the CM and SC groups.

A non-randomized pilot study based in Texas, USA, found a statistically significant effect of CM on smoking abstinence. In a pilot RCT in Boston, MA, USA, financial incentives increased brief smoking abstinence and the rate of quit attempts. A comparative effectiveness trial conducted among PEH who were veterans in the USA, showed that mobile CM delivered through a smartphone app and telephone-delivered group CBT with optional pharmacotherapy for 3 months, predicted prolonged smoking abstinence at 6 months (OR=3.07; 95% CI: 1.02–9.20, p=0.04). However, at 12 months, groups were no longer statistically different.

#### Feasibility and acceptability

Regarding CM feasibility and acceptability, a pilot study conducted among PEH veterans found that mobile CM may reduce smoking as an add-on to NRT or bupropion. Bio-verified 7 day point prevalence of smoking abstinence was 50% at 4 weeks. At follow-up, bio-verified abstinence was 65% at three and 60% at 6 months post-intervention.

A qualitative study among PEH at a health clinic in San Francisco, CA, USA, reported that a patient-centered clinical trial including financial incentives, flexible visits, and community navigators increased the feasibility of smoking cessation. Participants described the normative experiences of smoking, co-occurring substance use, and the use of tobacco to relieve stress as barriers to quitting. Nevertheless, most were interested in the proposed clinical trial and attempted to quit smoking. A qualitative study based on an RCT conducted in San Francisco, CA, USA, showed that participants viewed financial incentives as positive to reinforce smoking cessation and improve quality of life.

#### Secondary analyses

Secondary analyses of the RCT on CM showed that participants’ nicotine withdrawal symptoms persisted throughout the treatment period and increased in severity three to 4 weeks after the quit date. This suggests that targeting nicotine withdrawal symptoms could be relevant to help sustain long-term smoking cessation. Additionally, anticipatory withdrawal symptoms were common and linked to worse adherence to cessation aids. Another study showed that improvements in negative affect, restlessness, and stress, as well as positive coping expectancies, predicted tobacco quit date.

A study using cross-sectional and longitudinal data from a pilot RCT found that PEH who reported difficulties finding shelter, food, clothing, a place to wash, or go to the bathroom in the preceding month perceived more barriers to quitting smoking and were less likely to do so despite similar readiness, confidence, and quit attempts.

### Text messaging-based interventions (TMI)

The role of TMI in supporting smoking cessation, through an emphasis on social, economic, and family benefits, to help individuals develop adaptive strategies, increase motivation to quit, and remind them to use nicotine patches, was evaluated in three articles reporting on two studies. Two were pilot RCTs and one a randomized controlled study with cluster cross-over design. Two articles, reporting on the same study, included youths experiencing homelessness (YEH). Two articles also included a qualitative analysis. In all three studies, TMI was an add-on to weekly in-person counseling, smoking cessation group counseling, or another brief group counseling session. Both the TMI and the control groups received nicotine patches. Supplementary file Table 3 lists the characteristics of studies examining text messaging-based interventions to reduce smoking rates among PEH.

#### Evidence for smoking cessation outcomes

The effectiveness of TMI is mixed. The SmokefreeTXT intervention in drop-in centers serving YEH in Los Angeles, CA, USA, did not show statistically significant improvements in smoking abstinence. TMI showed potential for reducing smoking and increasing tobacco abstinence, although there were no statistically significant differences in continuous abstinence rates, measured four times per week following the tobacco quit date. While they steadily declined in both groups, the TMI condition consistently showed higher abstinence rates than the SC condition.

#### Feasibility and acceptability

Feasibility of TMI was not directly evaluated. Acceptability was generally high, with some variations related to the format and participant preferences. In Los Angeles, the proposed program was generally perceived as practical and well-structured: 86% of YEH offered to participate and complete it, indicating a high level of engagement. Participants in the pilot RCT with adult PEH (average age 45.6 years) preferred face-to-face interventions to SMS-only formats, which were considered impersonal and repetitive. Careful message design can improve acceptability, as suggested by the high approval rates of the SmokefreeTXT intervention conducted among adults in Boston, MA, USA.

### E-cigarettes

The effectiveness of e-cigarettes to stop or reduce smoking was examined in six studies reported in eight articles: one cluster RCT, one prospective four-center pragmatic cluster feasibility study with embedded qualitative process evaluation, one randomized pilot study, one non-RCT, one non-randomized feasibility trial, and one single-arm pilot study. Supplementary file Table 4 lists the characteristics of studies on e-cigarette interventions to reduce smoking rates among PEH.

#### Evidence for smoking cessation outcomes

Overall, the provision of e-cigarette starter kits to PEH showed mixed results in terms of smoking cessation, reduced cigarette consumption, and cost-effectiveness. A prospective four-center pragmatic cluster feasibility study with an embedded qualitative process evaluation conducted in two residential units and in 2 day centers in the UK, found that 6.3% of participants provided e-cigarettes achieved sustained abstinence at 24 weeks, compared to none in the control group. The e-cigarette group also showed a 43% reduction in the number of cigarettes smoked daily, versus 25% in the control group, although the percentage reduction in carbon monoxide levels was slightly higher in the latter. At follow-up, average healthcare and social expenses were consistently higher in the control than in the e-cigarette group.

In a subsequent cluster randomized controlled trial based on the same intervention, e-cigarette use did not result in sustained smoking abstinence after 24 weeks and was more costly than usual care.

A pilot study conducted in a day shelter in Oklahoma City, OK, USA, aiming to evaluate whether the addition of financial incentives might enhance the effectiveness of an e-cigarette intervention showed better uptake and a higher smoking abstinence rate.

In another non-randomized trial conducted in a supported temporary accommodation for the homeless in Ireland, self-reported reductions in cigarette consumption among participants who received e-cigarettes for 3 months were statistically higher than in the control group, although reductions in carbon monoxide measurements were not, and attrition was high.

The findings of a single-arm pilot study in Seattle, WA, USA, suggest that the combination of harm reduction counseling and e-cigarettes could effectively reduce nicotine dependence, smoking frequency, and intensity among PEH facing barriers to smoking cessation.

#### Feasibility and acceptability

While the feasibility of e-cigarette dispensation among PEH has not been explicitly examined, the acceptability of interventions promoting e-cigarette use seems high. Reports of e-cigarettes being lost, stolen, or exchanged were rare; some devices were broken, but most participants continued using either the e-cigarette they were provided with or a replacement one that they purchased. Participants’ perceptions of e-cigarettes appeared positive; they expressed interest in adopting e-cigarettes as an alternative to traditional smoking, and study retention rates were high, despite minor side effects, such as nervousness and headaches.

### Collaborative professional models

Four studies, reported in six articles, examined collaboration of professionals involved in delivering smoking cessation interventions with other staff in contact with PEH, of which three were based on a single-arm community-based uncontrolled trial, one was a pre-post-study, one was a cross-sectional survey, and one a qualitative study. Supplementary file Table 5 lists the characteristics of studies on collaborative and integrated professional models aiming to reduce smoking rates among PEH.

#### Evidence for smoking cessation outcomes

A single-arm community-based, uncontrolled trial tested a community pharmacist-linked cessation intervention in homeless shelters in San Francisco, CA, USA. Results suggest that a pharmacist-linked smoking cessation program in transitional homeless shelters can reduce structural barriers to cessation care and lower tobacco use. Residents engaged in the program increased quit attempts, but felt that to sustain abstinence, longitudinal tobacco treatment was needed. Adjusting for mental health conditions and use of other substances, the use of pharmacist-provided NRT was associated with lower weekly consumption and increased quit attempts.

An uncontrolled pre-post-study examined a smoking cessation treatment model delivered by the staff of a program for PEH in Melbourne, Australia, integrating nurse support with readily accessible cessation interventions (subsidized pharmacotherapy plus Quitline). While quit rates were low, treatment benefits included reduced tobacco consumption and butt smoking, significant financial savings, and psychological benefits (improved or stable mood).

#### Feasibility and acceptability

A cross-sectional survey evaluated the feasibility of implementing a pharmacist-led tobacco use screening and brief cessation intervention during mobile health access events across two food pantries and one homeless shelter in Indianapolis, IN, USA. Among participants who self-reported current use (29.6%), about half indicated readiness to quit smoking within 2 months; of these, nine out of ten accepted a tobacco Quitline card.

In a qualitative study of seven services dedicated to PEH who are US veterans, 54% of professionals reported delivering integrated care at the end of training and at 10 month post-follow-up. Providers rated face-to-face and collaborative team training as most helpful components. Barriers to collaborative learning included lack of leadership support and insufficient ability to electronically track progress through electronic medical records.

### Smoking cessation policies

Smoking cessation policies and practices in homeless shelters were examined in five studies reported in seven articles: two cross-sectional surveys, one pre-post-study examining a smoking ban intervention, the results of a questionnaire on smoking policies, and three qualitative studies. Three studies examined how service providers implemented smoking cessation support and drew elements of best practice. Supplementary file Table 6 lists the characteristics of studies on smoke-free and smoking cessation policies in services for PEH.

#### Evidence for smoking cessation outcomes

Findings from residents of a homeless shelter in San Diego, CA, USA, where smoking was prohibited indoors and outdoors within five blocks of the building, and permitted only four times during the day in designated smoking areas, suggest that smoke-free policies may not deter PEH with high smoking rates from attending shelters. Facility policies were also found to be associated with reduced smoking rates and increased quit attempts.

#### Feasibility and acceptability

Results of a qualitative study among residents of the homeless shelter studied in San Diego, CA, USA, suggest strong support for indoor and outdoor smoke-free policies to limit secondhand smoke exposure and an interest in e-cigarettes in areas of smoking prohibition. A US cross-sectional survey characterized PEH support (smokers, former smokers, and non-smokers) in terms of existing on-property smoke-free policies in a large homeless shelter in Dallas, TX, USA. The implementation of smoke-free spaces enjoyed more support from residents, particularly non-smokers, than initially anticipated by administrators.

A pre-post-intervention study examined resident support and expected/actual effects of a smoking ban in the same Dallas homeless shelter (before and after a partial outdoor smoking ban). Most participants supported the implementation of a large smoke-free zone in and around the shelter, but shelter-wide smoking bans were less positively viewed.

#### Good practice for service providers

A cross-sectional survey of homeless service providers documented smoking policies and cessation programs in 99 homeless services in the UK and identified areas of good practice and possible improvements. Smoking cessation support was not routinely offered to residents. Most centers did not provide staff training in smoking cessation. Areas of good practice included regular smoking cessation support embedded in routine health reviews or visits from Stop Smoking Services (SSS) offering tangible harm reduction support.

A qualitative study evaluating tobacco control policies in nine homeless housing programs in the US showed that although most programs had adopted smoke-free grounds, none of them assessed residents’ smoking status nor offered smoking cessation programs. In another qualitative study evaluating smoking cessation programs for YEH in the US, most of the 23 surveyed facilities expressed interest but did not offer smoking cessation services. Barriers to implementing formal smoking cessation programs included a lack of resources (e.g. money, personnel), staff training, and concern that smoking cessation may not be a high priority for youths. In this study, service providers seemed to prefer a less intensive smoking cessation program that could be delivered by staff.

## Discussion

### Main findings

This scoping review, based on 55 articles, shows that among PEH, different types of strategies promoting smoking cessation seem feasible and well-accepted, especially psychosocial support including counseling, motivational interviewing and contingency management. However, they show limited to moderate effectiveness. Similarly, text messaging demonstrates some effectiveness and is well-accepted, specifically regarding NRT access and adherence; however, we found no evaluation of the feasibility of this type of intervention. Provision of e-cigarettes appears very well accepted but shows limited effectiveness in terms of smoking reductions or quitting. Finally, collaborative professional models for smoking cessation promotion and smoking-related policies in homeless shelters seemed well-accepted; however, the level of evidence regarding their effectiveness was less robust.

### Adapting individual smoking cessation approaches to PEH

Most interventions aiming to promote smoking cessation among PEH combined pharmacotherapy and psychosocial components. In most studies included in this review, NRT was provided as part of SC. But SC varied considerably in terms of the type of medication, length of administration, and behavioral care. Importantly, the way in which psychosocial components were delivered was also heterogeneous. Financial incentives for smoking abstinence were considered feasible and acceptable; however, their relatively small positive effects diminished after the study period, raising questions about the sustainability of interventions primarily based on external motivating factors. The cost-effectiveness of CM was not investigated in studies included in this review. Overall, patient-centered and harm reduction-oriented approaches (involving counseling and e-cigarettes) were preferred by participants and supported by service providers. Patient-centered components – such as the ones implemented in the PTQ trial – were particularly appreciated and led to better smoking cessation outcomes. This is consistent with the promising effects of patient-centered care with regard to alcohol and drug recovery^21^. Interventions that garnered most support from PEH were those adapted to their needs, for example, flexible appointments, multiple reminders, individual rather than group counseling, and face-to-face rather than message-based-only motivational components. The feasibility of individually targeted smoking cessation interventions in this marginalized group depends on the ability to adapt to PEH-specific needs and the daily challenges they face. While counseling-based interventions may not always yield statistically significant results, tailoring them to emotional and social factors could enhance their effectiveness, particularly in the early phases of smoking cessation.

### Structural approaches to promote smoking cessation

Implementing structural smoking cessation interventions shifts the burden from PEH staff to staff of homeless shelters and other dedicated services. In several studies, shelter staff, nurses, and pharmacists were successfully trained to deliver smoking cessation interventions and provide brief advice. The integration of smoking cessation into regular activities was considered feasible and well accepted, provided it did not incur significant additional time commitments. Collaborative interventions among professionals with different expertise also showed promise. Smoke-free policies in homeless shelters were associated with reduced smoking rates and were supported by PEH, with large smoke-free zones generally favored over shelter-wide smoking bans.

### Comparisons with the general population and other marginalized groups

In the general population, pharmaceutical and behavioral interventions have been found to increase smoking cessation, whereas data for text messaging interventions are unclear^22^. Using a combination of behavioral support and pharmacotherapy increases the odds of successfully quitting smoking and sustaining smoking cessation at 6 months^23,24^. The results among PEH who smoke seem aligned with the general population, and further tailoring interventions may not be warranted. In particular, the provision of contingency management schemes among PEH may not necessarily be conducive to sustained abstinence. In other disadvantaged groups, results are similar. A scoping review^25^ on opportunistic smoking cessation interventions in people accessing financial support settings, nearly half of which were conducted in homeless support services, suggests that the financial benefit of quitting may be an important motivator among persons who experience disadvantage; the setting in which they receive financial support may be adequate to provide very brief smoking cessation advice and referral to evidence-based services. Nevertheless, the feasibility of smoking cessation programs remains essential and an issue that cannot be treated in the same way as in the general population.

Interventions examined in this scoping review were conducted in a variety of settings providing services to PEH, mostly emergency and transitional housing shelters, some specifically for youths and young adults, as well as veteran healthcare services and large-scale community events attended by PEH. This is because homelessness covers a range of situations, including sleeping rough or in temporary accommodation. Smoking cessation programs were implemented by a variety of staff with specific expertise, both medical and social. In particular, pharmacists and community physicians may be in a good position to implement innovative interventions among PEH. Extending the settings and types of staff developing smoking cessation interventions may broaden access to smoking cessation options for PEH and possibly help reach more persons motivated to engage in smoking cessation efforts.

One of the specificities of PEH is that they tend to accumulate multiple barriers reducing the odds of smoking cessation. For instance, there is evidence that among PEH, depression influences NRT adherence. Use of psychoactive drugs may also play a role. Nevertheless, in a single-arm community-based study, pharmacist-furnished NRT was associated with lower weekly consumption and increased quit attempts even after adjusting for mental health conditions and psychoactive substance use, suggesting that comorbidities do not entirely preclude smoking cessation.

### Strengths and limitations

Our study has some limitations that need to be discussed. Firstly, this scoping review does not have all the characteristics of a systematic review, namely that it did not include a quality assessment of selected studies. Secondly, smoking cessation interventions included in our review were set in English-speaking countries, mostly the United States. This calls for data from other contexts, where PEH and healthcare delivery options are different. In particular, there is a need for data on smoking cessation interventions from countries with sizeable numbers of PEH and universal healthcare settings, including Europe or low- and middle-income countries. Thirdly, we found only six studies on e-cigarette use, with only one RCT showing low effectiveness, yet deemed acceptable by PEH who smoke. Testing the use of e-cigarettes among PEH may be particularly relevant in the UK and Ireland, which have established-harm-reduction approaches. However, there is a need for further controlled studies integrating e-cigarettes within a broader range of smoking cessation treatments and strategies. Fourthly, a publication bias may exist, where negative or non-significant results would not have been published on the issue at hand. Finally, studies included in our review were heterogeneous, precluding the possibility of conducting a meta-analysis. Further studies in all intervention categories testing ways of increasing smoking cessation rates among PEH are needed to produce robust synthetic evidence. In particular, SMART trials that allow adaptations of standard cessation treatment may be beneficial.

Our study also has strengths that need to be highlighted. Firstly, PEH are among populations who experience the highest rates of smoking, and our scoping review adds to current knowledge regarding propitious ways of encouraging reductions in smoking rates in this marginalized group. Secondly, our review of the scientific literature can help service providers and healthcare professionals implement smoking cessation strategies that may truly help PEH.

## Conclusions

Psychosocial interventions, sometimes including contingency management, show limited to moderate effectiveness, and while well accepted, their feasibility and sustainability may be questionable – especially CM. Other types of support, above and beyond provision of NRT, have not been found to be effective. Additional studies are needed to identify effective, feasible and acceptable smoking cessation interventions among PEH. A possible way forward could be to combine individual and structural approaches and test interventions based on individuals’ preferences for smoking cessation strategies.
