# Supplementary material for: Feasibility and acceptability of strategies aiming to increase smoking cessation rates among people experiencing homelessness: A scoping review
Source: Tob Prev Cessat. 2026 Oct 1;12:10.18332/tpc/236153. doi: 10.18332/tpc/236153 (PMC13629338; doi:10.18332/tpc/236153)
Supplement: Supplementary file 1 [file TPC-12-58-s001.pdf]

TOBACCO PREVENTION AND CESSATION

Supplementary file

© 2026 Redonnet B. et al.

DOI:
10.18332/tpc/236153

The content has been provided by the author(s) and has not been reviewed, verified, or endorsed by European Publishing. It may not have undergone peer review. The views, opinions, and recommendations expressed are solely those of the author(s) and do not necessarily reflect the position of European Publishing. European Publishing accepts no responsibility or liability for any consequences arising from the use of, or reliance on, this content.

Supplementary Table 1. Characteristics of studies evaluating Counseling/Motivational Interviewing (MI) to reduce smoking rates among people experiencing homelessness

N= 21 out of 55 total selected articles. ~~Searched~~ Pubmed and Web of Science databases searched up to July 1st, 2026.

**Author, year**	**Country**	**Study Design**	**Participants**	**Sample size**	**Age**	**Aim**	**Intervention**	**Main findings**
Okuyemi et al., 2006	USA	Pilot RCT (PTQ)	18 years or older, >=5 cigarettes/day, smoked >=1 year, interested in quitting in the following 2 weeks, homeless.	46	43.8 (MI targeting smoking only);43.7 (MI targeting smoking, addictions and life events).	RCT of two MI approaches, assessment of the feasibility of a community-based smoking cessation intervention among homeless smokers.	Five individual MI sessions, six group meetings, and choice of 8 weeks of 21-mg nicotine patch or 4-mg nicotine lozenge. MI targeted either smoking behaviors exclusively or smoking and other addictions or life events that could affect ability to quit (smoking plus).	Results support the feasibility of conducting an MI smoking cessation intervention among homeless smokers. Findings also show promising effects for nicotine replacement therapy and counseling.
Shelley et al., 2010	USA	Single-arm pilot study	Current smokers, sheltered homeless persons	58	47	To test the effectiveness and feasibility of a smoking cessation intervention among sheltered homeless persons.	12-week smoking cessation intervention combining group MI and CBT plus pharmacotherapy. Examination of the effects of the intervention on smoking abstinence rates.	Results support the feasibility of enrolling and retaining sheltered homeless persons in a smoking cessation program. Counseling plus pharmacotherapy options may be effective in helping sheltered homeless smokers quit.
Goldade et al., 2011	USA	RCT (PTQ)	Participants residing in eight homeless shelters and transitional housing units.	430	N/A	To test the efficacy of MI to enhance adherence to nicotine replacement therapy (NRT) and smoking cessation outcomes.	8 weeks of NRT + 6 sessions of MI or control (NRT + SC) care. Six in-person assessment sessions and eight retention visits over 6 months.	The protocol is feasible, with the following elements: 1) a Community Advisory Board to engage participants; 2) location of the study in specific shelters to enhance visibility and access; 3) minimization of exclusion criteria; 4) adapting the calendar of study visits; 5) regular and persistent calls to remind participants of upcoming appointments using cell phones and shelter-specific channels of communication.
Bonevski et al., 2012	Australia	Single-group pre–post, proof of concept study.	English-speaking, receiving accommodation support from a homelessness	12 completed baseline measures	18 to 49	To explore the feasibility and acceptability of a low-cost, telephone-delivered program.	Six sessions once per week to provide personalized counseling about smoking cessation or reduction, as well as fruit and vegetable consumption, alcohol	High acceptability of the Phone for Health program judged convenient, useful and practical to help meet smoking reduction goals.

			outreach center.				use, physical activity and sun protection.	
Goldade et al., 2013	USA	Secondary analysis of RCT (PTQ)	Homeless smokers in emergency shelters and transitional housing units.	428 (324 returned at 26 week follow-up)	44.34	To examine the impact of knowing quitters on cessation after participation in an RCT.	Secondary analysis of data derived from the community-based RCT PTQ (intervention arm (NRT +MI) or control arm (NRT + standard care counseling).	Smoking cessation more likely in smokers who knew ≥ 5 quitters compared with those who knew no quitters.
Okuyemi et al., 2013	USA	RCT (PTQ)	Homeless smokers from emergency shelters and transitional housing units.	430 (216 MI; 214 Control)	44.4	To assess the effects of adding MI to nicotine patch for smoking cessation.	SC + six individual sessions of MI counseling vs. SC (8-week treatment of 21 mg nicotine patch) + one session of brief advice to quit smoking.	No difference in verified 7-day smoking abstinence rate at week 26 nor in the number of cigarettes smoked between baseline and week 26.
Reitzel et al., 2014	USA	Secondary analysis study of RCT (PTQ).	Homeless smokers in emergency shelters and transitional housing units.	22 (of 57 participants enrolled in the original study)	51,1	To explore associations between shelter proximity and real-time affect during specific smoking cessation attempt.	40 minutes weekly group cessation counseling sessions + NRT if prescribed by the on-site physician.	Proximity to the shelter cued negative affect and stress, due to the increased exposure to cigarettes and other smoking cues nearby. All participants relapsed to smoking by one week post-quit attempt.
Richards et al., 2015	USA	Secondary analysis of RCT (PTQ)	Homeless smokers, recruited from emergency shelters and transitional housing units.	430	44.35	To describe strategies to increase retention in a smoking cessation RCT.	Visits in locations convenient for participants; collection of different forms of contact information; flexible schedule with frequent low-intensity visits; incentives.	Overall retention of 75% at 26 weeks. Increased retention associated with older age; healthcare coverage; history of homelessness, low stress; high PHQ-9. Decreased retention associated with a history of alcohol and drug use.
Ojo-Fati et al., 2016	USA	Secondary analysis of RCT (PTQ)	Homeless smokers.	430	44.4	To identify predictors of adherence to NRT 8 weeks after randomization.	6 MI sessions + 8 weeks of NRT or SC (Brief quitting advice + 8 weeks of NRT).	NRT adherence associated with depression and motivation to quit.
Robinson et al., 2016	USA	Secondary analysis of RCT (PTQ)	Homeless smokers.	428	44.3	To assess the impact of depression symptoms (DS) on	MI or SC, with NRT for both arms. DS or control group using PHQ -9.	No effect of DS on smoking abstinence at week 8 or week 26. No significant interaction

				(173 DS, 255 Control)		smoking abstinence and the effect of MI at 26 weeks.		between depression symptoms (DS vs. Control) and the intervention (MI vs. SC).
Santa Ana et al., 2016	USA	Pre-post intervention	Homeless veterans addicted to alcohol and nicotine with co-morbid psychiatric conditions.	37<br>(16 GMI,<br>21 T-GMI)	GMI: 51.1<br>T-GMI: 54.5	Group MI including tobacco-specific components (T-GMI).	GMI to enhance engagement in substance abuse treatment and reduce substance use.<br>T-GMI protocol with additional content specific to cessation of tobacco use and enhancing smoking cessation treatment, in addition to the standard substance abuse content of GMI. 4 sessions over 4 consecutive days.	T-GMI participants more likely to attend tobacco cessation as well as combined tobacco cessation program with prescribed NRT.
Vijayaraghavan et al., 2016	USA	Pre-post-intervention	Current and former-smokers living and working in homeless shelters.	12 staff and 46 homeless persons	Site 1:<br>Staff: 48.8<br>Homeless: 38.4<br>Site 2:<br>Staff: 36<br>Homeless:45.8	To test the feasibility and acceptability of a capacity building intervention up to 12 weeks follow-up.	3.5 hours training for shelter staff in smoking cessation counseling.	Staff knowledge and efficacy in treating tobacco dependence increased. Acceptance of tobacco-related program services increased significantly among homeless persons from post-intervention to follow-up.
Ojo-Fati et al., 2017	USA	Report on RCT (PTQ1, PTQ2)	Homeless smokers.	N/A	N/A	Lessons learned based on two RCTs (PTQ 1 & 2).	Implementation of two RCTs: lessons learned, barriers and challenges.	The study shows challenges in smoking cessation for homeless persons: need to negotiate daily access to food, clothing and shelter.
Vijayaraghavan et al., 2019	USA	Community Intervention (non RCT)	Homeless adults.	52<br>(45 counseling; 7 counseling + pharmacotherapy)	N/A	To test an intervention introducing smoking cessation services in community day or service events; raise awareness and increase engagement with primary care.	Smoking cessation resources, behavioral counseling and pharmacotherapy provided to event attendees: 1) 2-week supply of NRT; 2) coupon for a free 4-week smoking cessation class at the UCSF Fontana Treatment Center; 3) a “quit kit” from San Francisco Department of Public Health; 4) a 1–800-NO-BUTTS card; 5) list of local tobacco cessation resources; 6) soft mints offered to who expressed interest in quitting.	2 participants out of 52 abstinent after the program.
Pratt et al., 2019	USA	Qualitative study	Homeless people	40	50.20	To understand the interplay between	One-on-one semi-structured interviews after the intervention	Participants described feeling pressure to smoke and drink in and around shelters and

			participating in an ongoing RCT (PTQ2) for smoking cessation.			the social environment and smoking cessation.	describing how the social environment impacts smoking cessation attempts and participant perceptions.	feeling motivated to quit and seeing smoking cessation as positive. Many participants more interested in reducing rather than quitting smoking.
Savoy et al., 2021	USA	Pre-post intervention	Homeless smokers recruited in a shelter.	57	48.8	Ecological Momentary Assessment (EMA) reporting affective variables on smoking behaviors following a specific quit attempt.	6 tobacco cessation classes using an American Cancer Society-based therapy and support group with NRT.	Affect and CPD significantly associated in the early part of the week but effects progressively fading. Increases in positive affect post- quit week significantly predicted fewer CPD.
Bhattacharya et al., 2022	USA	Secondary analysis of RCT (PTQ)	Homeless smokers enrolled in the 26-week RCT (PTQ) targeting smoking cessation.	324	45,3	To assess predictors of smoking reduction and quitting.	Data from PTQ RCT (MI+NRT vs brief advice to quit (SC)+NRT).	Higher tobacco use associated with lower reductions in smoking levels. Tobacco quitting vs. reduction more likely among men, persons experiencing housing instability, attached to quitting, with higher NRT adherence and lower illegal drug use.
Odukoya et al., 2022	USA	Survey to RCT participants (PTQ)	Homeless smokers.	315	45.7	To assess opinions on the Power to Quit study.	Participants enrolled in community-based RCT PTQ completed a 26 week- study feedback survey. Program satisfaction was measured by a single question on a 5-point Likert scale.	Visa gift Cards were the preferred incentive followed by bus tokens and Subway Restaurant coupons. NRT and counseling considered as helpful; phone calls or messages rated as helpful for appointment reminders. Majority very satisfied with the program schedule and program staff.
Pratt et al., 2022	USA	Qualitative study (PTQ2)	Homeless smokers.	40	50,20	Participants' experiences with the RCT intervention PTQ2 to inform future implementation .	Three-groups: 1) SC for smoking and alcohol cessation; 2) Intensive smoking cessation + SC alcohol abstinence counseling (IS); 3) Integrated Intensive Smoking and Intensive Alcohol Counseling (IntS+A). Counseling used individual CBT approach to smoking cessation and alcohol abstinence. 12 weeks of NRT for all participants. Qualitative semi-structured interviews, 25 in	Positive description of the PTQ2. Counseling and NRT products appreciated. Bond with PTQ2 staff and trust for emotional support and encouragement. The culture of alcohol use and cigarette smoking around the shelter environment presented a serious challenge.

							intervention group and 15 in control group.	
Skelton et al., 2022	Australia	Pilot study, pre-post-intervention	Male smokers attending a health clinic within a homelessness service.	20	44	To test the feasibility of providing varenicline in combination with NRT and MI.	12 weeks of intervention: 4 weeks of medication and NRT on 3 occasions. Weekly individual counseling sessions of 5-10 min, employing MI techniques (face to face or phone counseling).	65% retention 12-weeks post-intervention. 15%: reported continuous abstinence. 10% reported 30-day point prevalence abstinence, confirmed by CO level. 10 participants who did not quit smoking reported a significant reduction in the number of cigarettes smoked per day. Good adherence to pharmacological interventions. Low adherence to counseling sessions. NRT and MI rated highly acceptable. Some expressed concerns about the safety of varenicline.
Onigbogi et al., 2025	USA	RCT	PTQ2	344	46.8	To test the efficacy of combined smoking cessation and alcohol abstinence biobehavioral intervention, Intensive Smoking plus Alcohol (IS+A), versus SC for adults experiencing homelessness.	IS+A (10 sessions of cognitive behavioral therapy for smoking and alcohol cessation plus nicotine replacement therapy [NRT], n=168) or SC (educational session on smoking and alcohol cessation plus NRT, n=176).	At week 26, the IS+A intervention group did not differ from the SC group in expired carbon monoxide-verified 7-day point-prevalent smoking abstinence (16.6% vs. 12.8%, P=0.47) or rate of self-reported 30-day alcohol abstinence (91.1% vs. 90.2%, P=0.75). The IS+A intervention did not result in significantly better smoking or alcohol cessation outcomes than SC. Trends in smoking outcome data favored the intervention group.

Notes: CBT : Cognitive behavioral therapy, CO: Carbon monoxide, CPD: Cigarettes smoked per day, ENDS/EC: Electronic Nicotine Delivery Service/E-cigarette, N/A: Not available, NRT: Nicotine replacement therapy, PEH: People experiencing homelessness, PTQ: Power to Quit, RCT: Randomized controlled trial, SC/UC: Standard Care/Usual Care.
Age is expressed in mean years or a year range, unless indicated otherwise (median, average).

Supplementary Table 2. Characteristics of studies on contingency management (CM) and financial incentives to reduce smoking rates among people experiencing homelessness

N= 10 out of 55 total selected articles. ~~Searched~~ Pubmed and Web of Science databases searched up to July 1st, 2026.

**Author, year**	**Country**	**Study Design**	**Participants**	**Sample size**	**Age**	**Aim**	**Intervention**	**Main findings**
Businelle et al., 2014, a.	USA	Non-RCT	Homeless smokers in a shelter.	68<br>(58 SC,<br>10 SC+CM)	49,2	To compare the effectiveness of an adjunctive CM intervention plus small financial incentives vs. SC.	SC (weekly support groups + NRT if prescribed) vs SC + CM (financial incentives). 5 weeks (1 pre-quit + 4 post-quit). CM participants could earn a $20 gift card for biochemically-verified abstinence on the quit date. Escalating reinforcement schedule: gift card increased by $5 with each consecutive week of abstinence (40$). Non-abstinent participants also able to earn incentives if abstinence criteria met.	Significant effect of CM group on smoking abstinence (50% vs. 19% abstinent) and at 4 weeks post-quitting (30% vs. 1.7% abstinent).
Businelle et al., 2014, b.	USA	Secondary analysis study of non RCT (Businelle et al., 2014 a).	Homeless individuals recruited from a smoking cessation clinic in a homeless shelter.	57	N/A	To examine pre-cessation parameters linked with smoking abstinence.	Real-time assessment methodology used during 6 days prior to a scheduled quit attempt. Multiple variables (i.e., motivation for quitting, smoking expectancies, quit self-efficacy, smoking urges, negative affect, positive affect, restlessness, hostility, and stress) used as predictors of biochemically verified quit date abstinence.	Declining (negative slope) negative affect, restlessness, and stress predicted quit date abstinence. Increasing positive coping expectancies across the pre-quit week predicted quit date abstinence.
Carpenter et al., 2015	USA	Pilot	Homeless smokers	20	54.7	To evaluate a smart phone based application for CM named mobile CM	4 smoking cessation counseling sessions, nicotine replacement and bupropion (if medically eligible). 7 laboratory visits (set quit date after	Bioverified smoking abstinence of 60% at six months.

			between ages of 18–70.			(mCM), building on a web-based CM approach.	session 3), 1 brief telephone session, and 5 weeks of CO monitoring.	
Baggett et al., 2018 a.	USA	Pilot RCT	Homeless smokers	50 (25 intervention, 25 control)	46.6	To determine whether smoking in homeless persons is sensitive to financial rewards.	Escalating rewards ($15–$35) for brief smoking abstinence assessed 14 times over 8 weeks. Control arm given $10 at each assessment regardless of abstinence. All participants offered nicotine patches and counseling.	Financial incentives increased brief smoking abstinence and quit attempts at 8 weeks. Incentive arm participants made more quit attempts. Nicotine patch use and counseling attendance not significantly different between study groups. Alcohol and drug use did not change significantly.
Baggett et al., 2018 b	USA	Cross-sectional survey and longitudinal data from pilot RCT	Homeless smokers.	306 survey and 75 for pilot RCT	Survey : 47.6<br>RCT : 46.4	To examine associations between subsistence difficulties and 1) smoking cessation readiness, confidence, and barriers in a cross-sectional study, and 2) smoking abstinence during follow-up in a longitudinal study.	Cross-sectional data used to examine the association between subsistence difficulties and smoking cessation readiness, confidence, and barriers. Longitudinal data from an 8-week pilot RCT used to test the association between baseline subsistence difficulties and biochemically-defined smoking abstinence during follow-up.	Homeless smokers with greater subsistence difficulties perceive more barriers to smoking cessation. Greater subsistence difficulties are associated with more perceived barriers to quitting but not with cessation readiness or confidence. Dose-response relationship for most barriers, particularly psychosocial. Individuals with the highest level of subsistence difficulty had one-third the odds of being abstinent during follow-up compared to those without subsistence difficulties.
Rash et al., 2018	USA	RCT	Homeless smokers recruited from local homeless facilities.	70<br>(33 SC, 37 SC+CM)	SC : 45.5<br>SC+CM: 44.7	To examine smoking-related outcomes of homeless smokers receiving SC for smoking cessation with or without CM.	SC (smoking cessation with NRT, standard counseling and CO monitoring) vs SC + CM (behavioral intervention based on reinforcement in prizes, for negative CO sample submissions).	CM efficacious to increase initial quit rates in homeless smokers, significantly longer duration of abstinence and higher proportion of CO-negative samples relative to SC group. 4 weeks post-quit day: 22% in the CM condition abstinent, 9% in SC condition. At 6-months follow-up: about 10% in both conditions abstinent.

Sharbin et al., 2022	USA	Secondary data analysis of RCT (Rash et al., 2018)	Smokers experiencing homelessness.	70	45.07	To characterize nicotine withdrawal symptoms, during a quit attempt in treatment-seeking smokers experiencing homelessness.	Minnesota Nicotine Withdrawal Severity Index to assess symptoms three times prior to the scheduled quit date and twice weekly for 4 weeks post–quit date. Treatment outcomes: quit day smoking status, patch adherence, duration of abstinence, and percent negative CO samples.	Endorsement of withdrawal symptoms highest prior to the quit date and decreased over time with increases at weeks 3 to 4 (20% to 35%). Anticipatory withdrawal inversely related to nicotine patch adherence (p = .01), but not to abstinence-based treatment outcomes (p s > .05).
Miller et al., 2023	USA	Qualitative study	Current smokers, with current or past experiences of homelessness.	26	Median: 48.5	To explore views towards smoking cessation and explore factors influencing acceptability of engaging in a contingency management clinical trial.	Questionnaire and in-depth semi-structured interviews on tobacco use behaviors, prior cessation experiences, attitudes toward cessation, attitudes toward engaging in a clinical trial for cessation, and factors that might influence participation in a proposed contingency management clinical trial.	Most participants attempted to quit smoking and were interested in engaging in a clinical trial as a method to quit smoking.
Wilson et al., 2023	USA	Comparative effectiveness trial	Homeless smokers in VA healthcare, willing to make a quit attempt in the following 30 days.	127	55.72 for SC; 53.76 for mCM.	To assess the effectiveness of mCM, telephone- delivered CBT, tobacco cessation, pharmacotherapy, and long-term incentives.	mCM treatment (4 weeks of mCM, 5 weeks of telehealth counseling, and the option of 12 weeks of pharmacotherapy) or VA SC (3 biweekly group sessions and clinically appropriate pharmacotherapy). All participants randomly assigned to a $100 longer-term financial incentive for abstinence at 3-months follow-up; follow-up up to 12-months.	mCM cost-effective for smoking cessation among veterans experiencing homelessness. At 6-months, mCM associated with prolonged abstinence compared to SC (OR = 3.07, 95% CI [1.02, 9.20], p=0.04). By 12-months, no statistically significant group difference in smoking abstinence.
Gu et al., 2026	USA	Qualitative study of RCT	Adult PEH engaged in primary care.	Out of 83 participants in the RCT, 31 respondents to in-depth interview.	52	To examine experiences and perspectives on cessation among a subset of participants in a RCT of an extended (6-month) CM intervention for	The CM group received escalating incentives in the form of gift cards contingent on carbon monoxide-verified abstinence; the control group received fixed incentives for attending visits, not contingent on abstinence. All participants received	Participants experiencing homelessness viewed financial incentives as reinforcing smoking cessation and contributing to improved quality of life. Results of the parent clinical trial show that the extended CM intervention was effective in promoting 7-day point-prevalence abstinence at 6 months, and the CM group had significantly

						smoking cessation among PEH and who were engaged in primary care.	cessation care through their primary care clinics.<br>At one-year follow-up, in-depth interviews were conducted with a convenience sample (N = 31) from both study arms.	higher odds of achieving abstinence than the control group.

Notes: CBT : Cognitive behavioral therapy, CO: Carbon monoxide, CPD: Cigarettes smoked per day, ENDS/EC: Electronic Nicotine Delivery Service/E-cigarette, N/A: Not available, NRT: Nicotine replacement therapy, PEH: People experiencing homelessness, PTQ: Power to Quit, RCT: Randomized controlled trial, SC/UC: Standard Care/Usual Care.
Age is expressed in mean years or a year range, unless indicated otherwise (median, average).

Supplementary Table 3. Characteristics of studies examining Text Messaging-based Interventions (TMI) to reduce smoking rates among people experiencing homelessness

N= 3 out of 55 total selected articles. ~~Searched~~ Pubmed and Web of Science databases searched up to July 1st, 2026.

**Author, year**	**Country**	**Study Design**	**Participants**	**Sample size**	**Age**	**Aim**	**Intervention**	**Main findings**
Tucker et al., 2021	USA	Pilot RCT	Young people experiencing homelessness (YEH), current smokers desiring to quit, recruited from 3 drop-in centers.	77 (TMI:40)	18 to 25 years. Average : 22.6	To evaluate the feasibility of using a TMI for behavior change.	Group counseling + Nicotine patches vs. Group counseling session + Nicotine patches + TMI for 6 weeks. 3 to 6 automatic SMS per day to support quit attempt: amount of money saved; health and social benefits; managing cravings and negative moods; maintaining motivation; reminder to use nicotine patches.	6-week TMI support in addition to counseling promising for smoking reduction and abstinence among YEH smokers. Seven-day abstinence prevalence at 90-day follow-up higher than with SC. 90-day continuous abstinence rate not statistically different from zero in either condition. Reduction in the number of smoking days in the preceding 30 days from baseline to follow-up greater in TMI condition than in SC.
Linnemayr et al., 2021	USA	Randomized controlled study, cluster cross-over design	YEH, current smokers who desired to quit, recruited from 3 drop-in centers.	77 (TMI:40)	18 to 25 years. Average : 22.6	To describe the development of a smoking cessation TMI for homeless youths who are interested in quitting smoking.	Group-based cessation counseling session and nicotine patches, with random assignment to 6 weeks of TMI with additional smoking cessation support.	TMI was well accepted and feasible. Participant feedback points to the importance of carefully crafting text messages, leading to high (> 70%) approval of most text messaging components of the intervention. Participants enjoyed group counseling preceding TMI and suggested including more group elements into the intervention. Despite interruptions in phone use, participants reported being able to receive 95% of messages.
Baggett et al., 2019	USA	Pilot RCT	Homeless; >=18 years, >=100 cigarettes lifetime or >=5/day currently;	50	45.6	To assess whether TMI, added to evidence-based pharmacotherapy and counseling, improves smoking abstinence	8-week pilot RCT of nicotine patch therapy and weekly in-person counseling with (n=25) or without (n=25) TMI.	TMI, added to nicotine patch therapy and in-person counseling, did not significantly improve smoking abstinence.<br>56% participants (n=14) confirmed enrollment for ≥2 weeks. Smoking abstinence did not differ significantly between TMI and control arm participants. Of TMI enrollees who completed

			readiness to quit smoking; English proficiency.			among homeless adult smokers.		exit surveys (n=15), two-thirds were satisfied. Qualitative interviews (n=14) revealed that many participants preferred in-person over phone-based interventions, found TMI impersonal and robotic, and felt that messages were too frequent and repetitive.

Notes: CBT : Cognitive behavioral therapy, CO: Carbon monoxide, CPD: Cigarettes smoked per day, ENDS/EC: Electronic Nicotine Delivery Service/E-cigarette, N/A: Not available, NRT: Nicotine replacement therapy, PEH: People experiencing homelessness, PTQ: Power to Quit, RCT: Randomized controlled trial, SC/UC: Standard Care/Usual Care.
Age is expressed in mean years or a year range, unless indicated otherwise (median, average).

Supplementary Table 4 : Characteristics of studies on E-cigarette interventions to reduce smoking rates among people experiencing homelessness

N= 8 out of 55 total selected articles. ~~Searched~~ Pubmed and Web of Science databases searched up to July 1st, 2026.

**Author, year**	**Country**	**Study Design**	**Participants**	**Sample size**	**Age**	**Aim**	**Intervention**	**Main findings**
Collins et al., 2019	USA	Single-arm pilot study	Smokers experiencing chronic homelessness.	44	48.24	EC + smoking Harm reduction: a) participant-led tracking of smoking-related outcomes, b) harm-reduction goals and progress made toward them, c) relative risks of nicotine delivery systems, and d) instructions on use of safer nicotine products.	Group and individual sessions + NRT + ENDS.	Harm-reduction counseling plus ENDS shows promise for smokers experiencing chronic homelessness. Reductions in cigarette dependence, frequency and intensity. ENDS use associated with an additional 44% reduction in smoking intensity and a 1.2-point reduction in dependence. 24% and 27% weekly uptake in NRT and ENDS use, respectively. Each week associated with a 18% increase in odds of abstinence.
Dawkins et al., 2020	UK	Non-RCT, feasibility trial.	>=18 years, daily smokers, accessing homeless centre services.	80	42,66	To assess the feasibility of supplying free EC starter kits.	Supplying of free EC starter kits.	Reasonable recruitment and retention rates. Intervention well received with minimal negative effects and few unintended consequences (e.g. loss, theft, adding illicit substances).
Scheibein et al., 2020	Ireland	Non-RCT	People accessing a supported temporary accommodation (STA) homeless service	9 (14 out of 23 did not complete the intervention)	43.89	To explore the efficacy, challenges and opportunities of using ENDS to support cessation of tobacco smoking with people accessing STA homeless service.	Participants were given an ENDS for 3 months and two 10-ml bottles containing e-liquid available in several flavours and at several strengths, delivery on a weekly basis. Follow-up at 12 weeks.	Statistically significant self-reported reductions in cigarette consumption, nicotine dependence, but no reductions in carbon monoxide. Barriers to engagement were peer norms, vaping restrictions and adverse life events. Positive effects reported included increased energy, less coughing, better breathing and financial benefits.

Cox et al., 2022	UK	Prospective four-center pragmatic cluster feasibility study with embedded qualitative process evaluation.	Smokers accessing homeless centers (two residential units and two day centers).	80	42.66	To evaluate the offer of an ENDS or SC for smokers accessing a homeless center.	Single refillable EC provided together with e-liquid once per week for 4 weeks (three flavours; two nicotine strengths: 12 or 18 mg/ml) with information on EC use and information on quitting smoking.	Retention rates of 75%, 63% and 59% at 4, 12 and 24 weeks, respectively. High perceived value of the intervention. Barriers included participants' personal difficulties and cannabis use. Facilitators were desire to change, free electronic cigarettes and social dynamics. Good staff capacity to support the study.
Sifat et al., 2024	USA	Pilot study	Adults who smoked combustible cigarettes (CC) daily recruited from an urban day shelter	60	48.8	To determine whether switch to EC is a useful tobacco harm reduction approach and to evaluate the addition of financial incentives.	EC with or without financial incentives. All participants received an EC device and nicotine pods during for 4 weeks; those in the ENDS+FI group also received escalating weekly incentives for abstinence during the same period. Key follow-ups were conducted at 4- and 8-weeks post-switch.	EC switching with financial incentives promising as a harm reduction tool. CC smoking abstinence rates among ENDS and ENDS +FI were 3.3 % vs. 13.3 % at 4 weeks and 10.0 % vs. 13.3 % at 8 weeks in the control group. ENDS +FI participants reported fewer days of smoking, more days of EC use, and greater reductions in CO at 4-week follow-up. Most participants reported a high likelihood of switching to EC (67.7 %).
Dawkins et al., 2025	UK	Cluster RCT	Adults who smoked daily and accessed 32 homeless support centres	475 in 32 homeless support centres	43.7	To measure the effectiveness of an EC starter kit and 4-week supply of e-liquids provided to people who smoked accessing homeless support centres compared with usual care.	EC starter kit and 4-week supply of e-liquids provided in e-cigarette arm vs UC arm (brief advice and signposting to local stop smoking service).	EC did not support sustained smoking abstinence for 24 weeks. Sustained 24 weeks CO-validated smoking cessation rates were 5/239 (2.1%) with EC vs. 2/236 (0.8%) with UC (aRR: 2.43, 95%CI: 0.51–11.64)
Ford et al., 2025	UK	Process evaluation of Cluster RCT	Adults who smoked daily and accessed 32 homeless support centres	475 in 32 homeless centres	43.7	To explore the factors which influenced potential smoking abstinence among participants receiving an EC intervention within the cluster RCT.	EC starter kit and 4-week supply of e-liquids provided in e-cigarette arm vs usual care arm (brief advice and signposting to local stop smoking service).	After receiving the intervention, participants demonstrated high capability to use EC and appreciation of EC for harm reduction. Some participants reported dissatisfaction with vaping. Opportunity for behaviour change was strengthened by perceived acceptability to vape and social support beyond the centre but hindered by a smoking culture and perceived lack of staff support.

								Motivation was enhanced by EC efficacy belief, pride from cutting down, and financial benefits of vaping, but negatively impacted by challenging personal circumstances
Li et al., 2025	UK	Cluster RCT	Adults who smoked daily and accessed 32 homeless support centres	475 in 32 homeless centres	43.7	To determine the cost-effectiveness of providing an EC for smoking cessation in homeless support centres compared to usual care.	EC starter kit and 4-week supply of e-liquids provided in e-cigarette arm vs usual care arm (brief advice and signposting to local stop smoking service).	Providing EC for smoking cessation in homeless support centres was more costly than usual care, but the small increase in quality-adjusted life-years was not significant.

Notes: CBT : Cognitive behavioral therapy, CO: Carbon monoxide, CPD: Cigarettes smoked per day, ENDS/EC: Electronic Nicotine Delivery Service/E-cigarette, N/A: Not available, NRT: Nicotine replacement therapy, PEH: People experiencing homelessness, PTQ: Power to Quit, RCT: Randomized controlled trial, SC/UC: Standard Care/Usual Care.
Age is expressed in mean years or a year range, unless indicated otherwise (median, average).

Supplementary Table 5. Characteristics of studies on collaborative professional models for interventions to reduce smoking among people experiencing homelessness

N= 6 out of 55 total selected articles. ~~Searched~~ Pubmed and Web of Science databases searched up to July 1st, 2026.

**Author, year**	**Country**	**Study Design**	**Participants**	**Sample size**	**Age**	**Aim**	**Intervention**	**Main findings**
Segan et al., 2015	Australia	Uncontrolled before and after study.	Individuals experiencing homelessness interested in reducing smoking or quitting, 18 year old or above; nurses.	49 enrolled<br>14 nurses	Average : 48	To examine the feasibility, acceptability, and effectiveness of a smoking cessation treatment model delivered by staff of a homeless persons programme.	Co-management model involving a nurse, a doctor, and Quitline: nurse-assisted bulk-billed doctor appointment to review physical and mental health, medications affected by tobacco and prescription of nicotine patch and/or bupropion, or varenicline for up to 3 months; up to 12 weekly smoking cessation appointments with a nurse; proactive phone support for 3 months. Assessment 6 months post-enrolment.	Integrating nurse support with readily accessible cessation interventions was feasible and acceptable. While quit rates were low, treatment benefits included harm-reduction, significant financial savings, and psychological benefits. Tobacco expenditure and CO readings at follow-up approximately halved. Participants who used Quitline were more likely to report a 50% or higher reduction in tobacco consumption at 6 months.
Beckham et al., 2018	USA	Qualitative study.	Staff members from 7 Veteran Affairs (VA) homeless provider teams.	34	N/A	To evaluate the feasibility of an integrated care (IC) approach to improve smoking cessation interventions and evaluate the utility of the learning collaborative (LC) model.	Self-reported questionnaires, to assess providers' perceptions of the LC model and the number of providers delivering IC.	54% of providers reported delivering IC at the end of training and at 10-months. Providers rated face-to-face trainings and collaborative team trainings as the most helpful LC components. Barriers to the use of LC included lack of leadership support and lack of ability to electronically track progress through the electronic medical record.
Hartman-Filson et al., 2022	USA	Single-arm, community-based,	PEH and trained shelter staff.	PEH :52<br>Staff :69	50	To test a community pharmacy-linked smoking cessation program integrated	Shelter staff trained to provide brief cessation counseling, tested in two shelters including one-time pharmacist-delivered cessation	Community-based pharmacists judged effective in expanding smoking cessation services in homeless shelters.

		uncontrolled trial.				within two homeless shelters to increase quit attempts and reduce daily cigarette consumption.	counseling and nicotine replacement therapy (NRT) for 3 months.	70% of participants reported making a quit attempt, 84% used NRT. Having an encounter with staff in the past week was associated with a 40% reduction in weekly consumption.
De Los Reyes et al., 2023	USA	Single-arm community-based, uncontrolled trial.	Homeless persons recruited from three homeless shelters.	51	46.5	To evaluate a community pharmacist-linked cessation intervention.	One-time pharmacist-delivered counseling and NRT for 3months. Questionnaires at baseline and during 12 weekly follow-up visits. In-depth interviews with residents to understand barriers to and facilitators of engagement.	A pharmacist-linked smoking cessation program can reduce structural barriers to cessation care and reduce tobacco use among people experiencing homelessness: average daily cigarette consumption was reduced by over 55% CO-verified abstinence. Use of medications in preceding week associated with a 29% reduction in weekly consumption, and increased odds of a quit attempt (x2.89).
Hudmon et al., 2023	USA	Cross-sectional survey.	Participants recruited in 11 health access events across 2 food pantries and 1 homeless shelter.	639 individuals assessed for tobacco use.	N/A	To evaluate the feasibility of a pharmacist-led tobacco use screening and brief cessation intervention during mobile health access events.	Survey administered verbally by pharmacy team members as part of a broader intervention, which also involved the administration of vaccines (COVID-19 and influenza). Brief cessation counseling provided by pharmacy personnel.	Pharmacist-led health events at sites serving populations that are under-resourced affording unique opportunities to interface with and provide brief interventions for smokers. Among the 639 persons assessed for tobacco use, 189 self-reported current use (29.6%); 23.7% at food pantries, and 66.7% at their homeless shelter. About half indicated readiness to quit within 2 months; of these, 9 out of 10 accepted a tobacco quitline card.
Kui et al., 2025	USA	Single-arm community-based, uncontrolled trial.	adults experiencing homelessness from three homeless shelters	206	47	To explore the role of mental health and substance use disorders on cigarette consumption and weekly quit attempts among adults experiencing homelessness who were engaged in a community pharmacist-linked	12-week pharmacist-linked smoking cessation program that involved pharmacist counseling and furnishing of NRT and brief cessation coaching by trained shelter staff.	Adjusting for baseline mental health conditions and other covariates, the use of pharmacist-furnished NRT was associated with lower weekly consumption (Incidence Rate Ratio [IRR]: 0.74, 95 % Confidence Interval [CI]: 0.71–0.76) and increased weekly quit attempts (Adjusted Odds Ratio (AOR): 1.78, 95 % CI: 1.23–2.58). After adjusting for baseline substances used and other covariates, use of pharmacist-furnished NRT was associated with lower weekly consumption (IRR: 0.74, 95 % CI: 0.73–0.76) and increased weekly quit attempts (AOR: 1.99, 95 % CI: 1.45–2.74).

						smoking cessation program.		

Notes: CBT : Cognitive behavioral therapy, CO: Carbon monoxide, CPD: Cigarettes smoked per day, ENDS/EC: Electronic Nicotine Delivery Service/E-cigarette, N/A: Not available, NRT: Nicotine replacement therapy, PEH: People experiencing homelessness, PTQ: Power to Quit, RCT: Randomized controlled trial, SC/UC: Standard Care/Usual Care.
Age is expressed in mean years or a year range, unless indicated otherwise (median, average).

Supplementary Table 6. Characteristics of studies on smoke-free and smoking cessation policies in services for people experiencing homelessness

N= 7 out of 55 total selected articles. ~~Searched~~ Pubmed and Web of Science databases searched up to July 1st, 2026.

**Author, year**	**Country**	**Study Design**	**Participants**	**Sample size**	**Age**	**Aim**	**Intervention**	**Main findings**
Shadel et al., 2014	USA	Qualitative study	19 service providers of 23 shelters and drop-in centers serving homeless youths.	19 service providers	N/A	To document service providers' interest in smoking cessation services to homeless youths, potential barriers, and ways to overcome them.	No intervention. Perceptions of smoking cessation policies and implementation. Semi-structured telephone interviews with 19 service providers from 23 shelters and drop-in centers serving homeless youths.	91% of homeless facilities were interested in smoking cessation services but 84% did not offer any. Barriers included lack of resources (e.g., money, personnel) to support the programs, staff training, and concern that smoking cessation may not be a high priority for homeless youths. Service providers preferred less intensive smoking cessation program that could be delivered on-site.
Businelle et al., 2015	USA	Pre-post Intervention	Homeless adults	394	43.3	To examine guest support for and expected/actual effects of a smoking ban.	2 waves (before and after partial outdoor smoking ban).	Most participants were smokers (76%) and supported the creation of a large smoke-free zone, but support for a shelter-wide smoking ban was limited. Participants who completed both study waves experienced a reduction in expired carbon monoxide at wave 2.
Vijayaraghavan et Pierce. 2015	USA	Questionnaire after Intervention	Homeless adults in emergency and transitional housing facilities.	170	43,3	Current and former smokers were interviewed on smoking behaviors and attitudes toward smoking policies.	No intervention. Perceptions of smoke-free policies. Prohibition of smoking indoors and outdoors within five blocks of the building, permitted during four smoking breaks per day outside the building.	Smoke-free policies judged to reduce smoking behaviors. Three-fourths of smokers agreed that facility policies reduced their smoking, and about half thought they were associated with either a quit attempt or cessation. 60% agreed that further restrictions could increase interest in quitting smoking completely.
Sung et al., 2017	USA	Qualitative study	Administrators of 9 homeless service agencies.	N/A	N/A	To evaluate tobacco control policies in homeless housing programs	No intervention. Survey and open-ended interviews assessing institutional smoking- policies and cessation programs; barriers and	Most programs adopted smoke-free grounds and limited staff smoking. Most administrators receptive to policies that promote a tobacco-free culture; however, clients have unique challenges

							receptivity to tobacco control interventions.	making traditional smoking cessation programs unfeasible.
Vijayaraghavan et al., 2017	USA	Qualitative study	Homeless current or former smokers.	66	42.9	To examine attitudes toward smoke-free policies and perceptions of e-cigarette use.	No intervention. Perceptions of smoke-free policies and e-cigarette use, anti-tobacco norms, attitudes toward smoke-free policies.	Strong support for indoor and outdoor smoke-free policies to limit secondhand smoke exposure for non-smokers and children.<br>Interest in e-cigarettes in areas of smoking prohibition and/or as a cessation aid.
Cox et al., 2022	UK	Cross sectional survey including structured and open-ended questions.	Homeless services recruited from charities.	99 homeless centres	N/A	To document smoking and e-cigarette policies of UK homeless services and identify areas of good practice.	No intervention. Perceptions of smoke-free policies and smoking cessation support. 99 homeless services answered open-ended questions: policies, screening, cessation, training for staff, types of cessation support, good practice.	Smoking accounted for across different policy types (92%, 56% stand-alone smoking policy) and restricted in some areas (84% with some indoor and outdoor areas). Most centers did not provide staff training to support smoking cessation. Good practice included smoking cessation support embedded in routine health reviews or visits from SSS and tangible harm reduction support. Areas for improvement include staff training, staff smoking with service users and skipping routine screening questions around smoking.
Martinez et al., 2022	USA	Cross-sectional survey	Adult guests of a homeless shelter.	394	43.3	To characterize guest support for smoke-free policies within a homeless shelter.	No intervention. Perceptions of smoke-free policies. Assessment of participant sociodemographics, smoking status, behavioral health diagnoses, and support for two versions of an on-property outdoor courtyard smoke-free policy.	Implementation of smoke-free living/workplace policies in homeless shelters enjoy more support - specifically, non-smokers - than anticipated by shelter administrators. Overall, 64.0% of participants supported a partial, and 32.0% a full smoking ban. Older participants, non-veterans, former smokers, and those without severe mental illness had significantly higher odds of supporting a partial smoking ban. Relative to current smokers, never smokers and former smokers had significantly greater odds of supporting a complete smoking ban.

Notes: CBT : Cognitive behavioral therapy, CO: Carbon monoxide, CPD: Cigarettes smoked per day, ENDS/EC: Electronic Nicotine Delivery Service/E-cigarette, N/A: Not available, NRT: Nicotine replacement therapy, PEH: People experiencing homelessness, PTQ: Power to Quit, RCT: Randomized controlled trial, SC/UC: Standard Care/Usual Care.
Age is expressed in mean years or a year range, unless indicated otherwise (median, average).

Appendix 1: Complete citations for included articles (N=55)

Baggett T, McGlave C, Kruse G, Yaqubi A, Chang Y, Rigotti N. SmokefreeTXT for Homeless Smokers: Pilot Randomized Controlled Trial. *JMIR mHealth and uHealth*. 2019;7(6). doi:10.2196/13162

Baggett TP, Chang Y, Yaqubi A, McGlave C, Higgins ST, Rigotti NA. Financial Incentives for Smoking Abstinence in Homeless Smokers: A Pilot Randomized Controlled Trial. *Nicotine Tob Res.* 2018;20(12):1442-1450. doi:10.1093/ntr/ntx178

Baggett TP, Yaqubi A, Berkowitz SA, et al. Subsistence difficulties are associated with more barriers to quitting and worse abstinence outcomes among homeless smokers: evidence from t wo studies in Boston, Massachusetts. *BMC Public Health*. 2018;18(1):463. doi:10.1186/s12889-018-5375-z

Beckham JC, Kirby AC, Tise N, et al. A Learning Collaborative Model for Implementation of Smoking Cessation among Homeless Veterans. *J Smok Cessat*. 2018;13(3):129-136. doi:10.1017/jsc.2017.15

Bhattacharya M, Ojo-Fati O, Everson-Rose SA, et al. Smoking reduction among homeless smokers in a randomized controlled trial targeting cessation. *Addict Behav*. 2022;133:107373. doi:10.1016/j.addbeh.2022.107373

Bonevski B, Baker A, Twyman L, Paul C, Bryant J. Addressing smoking and other health risk behaviours using a novel tele phone-delivered intervention for homeless people: A proof-of-concept study. *Drug Alcohol Rev*. 2012;31(5):709-713. doi:10.1111/j.1465-3362.2012.00438.x

Businelle MS, Kendzor DE, Kesh A, et al. Small financial incentives increase smoking cessation in homeless smokers: A pilot study. Addictive Behaviors. 2014;39(3):717-720. doi:10.1016/j.addbeh.2013.11.017

Businelle MS, Ma P, Kendzor DE, et al. Predicting quit attempts among homeless smokers seeking cessation treatment: an ecological momentary assessment study. *Nicotine Tobacco Res*. 2014;16(10):1371-1378. doi:10.1093/ntr/ntu088

Businelle MS, Poonawalla IB, Kendzor DE, et al. Smoking policy change at a homeless shelter: Attitudes and effects. *Addict Behav*. 2015;40:51-56. doi:10.1016/j.addbeh.2014.08.013

Carpenter VL, Hertzberg JS, Kirby AC, et al. Multicomponent smoking cessation treatment including mobile contingency management in homeless veterans. *J Clin Psychiatry*. 2015;76(7):959-964. doi:10.4088/JCP.14m09053

Collins SE, Nelson LA, Stanton J, et al. Harm reduction treatment for smoking (HaRT-S): findings from a single- arm pilot study with smokers experiencing chronic homelessness. *Subst Abuse*. 2019;40(2):229-239. doi:10.1080/08897077.2019.1572049

Cox S, Ford A, Li J, et al. Exploring the Uptake and Use of Electronic Cigarettes Provided to Smokers Accessing Homeless Centres: A Four-Centre Cluster Feasibility Trial. NIHR Journals Library; 2021. Accessed February 18, 2025. http://www.ncbi.nlm.nih.gov/books/NBK570404/

Cox S, Murray J, Ford A, Holmes L, Robson D, Dawkins L. A cross-sectional survey of smoking and cessation support policies in a sample of homeless services in the United Kingdom. *BMC Health Serv Res*. 2022;22(1):1-10. doi:10.1186/s12913-022-08038-7

Dawkins L, Bauld L, Ford A, et al. A cluster feasibility trial to explore the uptake and use of e-cigarettes versus usual care offered to smokers attending homeless centres in Great Britain. *PLOS ONE*. 2020;15(10). doi:10.1371/journal.pone.0240968

Dawkins L, Soar K, Pesola F, et al. Smoking cessation for people accessing homeless support centres (SCeTC H): comparing the provision of an e-cigarette versus usual care in a cluster randomised controlled trial in Great Britain. *BMC Med*. 2025;23(1):394. doi:10.1186/s12916-025-04167-y

De Los Reyes G, Ng A, Valencia Chavez J, et al. Evaluation of a Pharmacist-Linked Smoking Cessation Intervention for Adults Experiencing Homelessness. *Subst Use Misuse*. 2023;58(12):1519-1527. doi:10.1080/10826084.2023.2231060

Ford A, McMillan L, Soar K, et al. Exploring how an e-cigarette intervention influenced tobacco smoking behaviour in people accessing homelessness services: Findings from the SCeTCH trial process evaluation. *Int J Drug Policy*. 2025;143:104901. doi:10.1016/j.drugpo.2025.104901

Goldade K, Whembolua G, Thomas J, et al. Designing a smoking cessation intervention for the unique needs of homeless persons: a community-based randomized clinical trial. *Clin Trials*. 2011;8(6):744-754. doi:10.1177/1740774511423947

Goldade K, Des Jarlais D, Everson-Rose SA, et al. Knowing Quitters Predicts Smoking Cessation in a Homeless Population. *Am J Health Behav*. 2013;37(4):517-524. doi:10.5993/AJHB.37.4.9

Gu D, Cuby J, Gomez O, et al. Money Helps: Financial Incentives for Smoking Cessation Among People Experiencing Homelessness. *Subst Use Misuse*. 2026;0(0):1-11. doi:10.1080/10826084.2026.2682312

Hartman-Filson M, Chen J, Lee P, et al. A community-based tobacco cessation program for individuals experiencing homelessness. *Addict Behav*. 2022;129:107282. doi:10.1016/j.addbeh.2022.107282

Hudmon K, Czarnik J, Lahey A, et al. Pharmacist-Led Implementation of Brief Tobacco Cessation Interventions during Mobile Health Access Events. *Pharmacy*. 2023;11(2). doi:10.3390/pharmacy11020072

Kui K, Hailozian C, Thai M, et al. Tobacco cessation, mental health, and substance use in a community pharmacist-linked cessation program for people experiencing homelessness. Drug Alcohol Depend. 2025;267:112527. doi:10.1016/j.drugalcdep.2024.112527

Li J, Wu Q, Parrott S, et al. Cost-effectiveness of e-cigarettes for smoking cessation at homeless support centres: SCeTCH cRCT. Public Health Res. Published online November 12, 2025:1-45. doi:10.3310/GJLD2428

Linnemayr S, Zutshi R, Shadel W, Pedersen E, DeYoreo M, Tucker J. Text Messaging Intervention for Young Smokers Experiencing Homelessness: Lessons Learned From a Randomized Controlled Trial. *JMIR mHealth and uHealth*. 2021;9(4):e23989. doi:10.2196/23989

Martinez J, Jafry MZ, Chen TA, et al. Guest Support for Outdoor Smoke-Free Policies within a Homeless Shelter. *Int J of Environ Res Public Health*. 2022;19(4):2408. doi:10.3390/ijerph19042408

Miller J, Cuby J, Hall SM, et al. Tobacco use behaviors and views on engaging in clinical trials for tobacco cessation among individuals who experience homelessness. *Contemp Clin Trials Commun*. 2023;32:101094. doi:10.1016/j.conctc.2023.101094

Odukoya OO, Abdiwahab EA, Olubodun T, Azagba S, Ogunsola FT, Okuyemi KS. Implementing a smoking cessation intervention for people experiencing homelessness:

Participants' preferences, feedback, and satisfaction with the "power to quit" program. *PloS One*. 2022;17(6):e0268653. doi:10.1371/journal.pone.0268653

Ojo-Fati O, Thomas JL, Vogel RI, Ogedegbe O, Jean-Louis G, Okuyemi KS. Predictors of Adherence to Nicotine Replacement Therapy (Nicotine Patch) Among Homeless Persons Enrolled in a Randomized Controlled Trial Targeting Smoking Cessation. *J Family Med*. 2016;3(7):1079.

Ojo-Fati O, Joseph AM, Ig-Izevbekhai J, et al. Practical issues regarding implementing a randomized clinical trial in a homeless population: strategies and lessons learned. *Trials*. 2017;18:1-10. doi:10.1186/s13063-017-2046-9

Okuyemi KS, Goldade K, Whembolua GL, et al. Motivational interviewing to enhance nicotine patch treatment for smoking cessation among homeless smokers: a randomized controlled trial. *Addiction*. 2013;108(6):1136-1144. doi:10.1111/add.12140

Okuyemi KS, Thomas JL, Hall S, et al. Smoking Cessation in Homeless Populations: A Pilot Clinical Trial. *Nicotine Tob Res*. 2006;8(5):689-699. doi:10.1080/14622200600789841

Onigbogi O, Pratt R, Luo X, et al. Combined smoking cessation and alcohol abstinence treatment for people experiencing homelessness: a randomized trial. *Nicotine Tob Res: Off J Soc Res Nicotine Tob*. Published online May 16, 2025:ntaf085. doi:10.1093/ntr/ntaf085

Pratt R, Pernat C, Kerandi L, et al. "It's a hard thing to manage when you're homeless": the impact of the social environment on smoking cessation for smokers experiencing homelessness. *BMC Public Health*. 2019;19(1):635. doi:10.1186/s12889-019-6987-7

Pratt R, Xiong S, Kmiecik A, et al. The implementation of a smoking cessation and alcohol abstinence intervention for people experiencing homelessness. *BMC Public Health*. 2022;22(1):1-8. doi:10.1186/s12889-022-13563-5

Rash CJ, Petry NM, Alessi SM. A randomized trial of contingency management for smoking cessation in the homeless. *Psychol Addict Behav*. 2018;32(2):141-148. doi:10.1037/adb0000350

Reitzel LR, Kendzor DE, Nga Nguyen, et al. Shelter Proximity and Affect among Homeless Smokers Making a Quit Attempt. *Am J Health Behav*. 2014;38(2):161-169. doi:10.5993/AJHB.38.2.1

Richards CM, Sharif F, Eischen S, et al. Retention of Homeless Smokers in the Power to Quit Study. *Nicotine Tob Res*. 2015;17(9):1104-1111. doi:10.1093/ntr/ntu210

Robinson CD, Rogers CR, Okuyemi KS. Depression Symptoms Among Homeless Smokers: Effect of Motivational Interviewing. *Subst Use Misuse*. 2016;51(10):1393-1397. doi:10.3109/10826084.2016.1170143

Santa Ana EJ, LaRowe SD, Armeson K, Lamb KE, Hartwell K. Impact of group motivational interviewing on enhancing treatment engagement for homeless Veterans with nicotine dependence and other substance use disorders: A pilot investigation. *Am J Addict*. 2016;25(7):533-541. doi:10.1111/ajad.12426

Savoy EJ, Businelle MS, Nguyen N, et al. Examining moment to moment affective determinants of smoking rate following a quit attempt among homeless daily smokers. *Addict Behav*. 2021;115:106788. doi:10.1016/j.addbeh.2020.106788

Scheibein F, McGirr K, Morrison A, Roche W, Wells J. An exploratory non-randomized study of a 3-month electronic nicotine delivery system (ENDS) intervention with people accessing a homeless supported temporary accommodation service (STA) in Ireland. *Harm Reduct J*. 2020;17(1). doi:10.1186/s12954-020-00406-y

Segan CJ, Maddox S, Borland R. Homeless Clients Benefit From Smoking Cessation Treatment Delivered by a Homeless Persons' Program. *Nicotine Tob Res*. 2015;17(8):996-1001. doi:10.1093/ntr/ntv062

Shadel WG, Tucker JS, Mullins L, Staplefoote L. Providing smoking cessation programs to homeless youth: the perspective of service providers. *J Subst Abuse Treat*. 2014;47(4):251-257. doi:10.1016/j.jsat.2014.05.009

Sharbin GK, Rash CJ. Characterizing nicotine withdrawal in smokers experiencing homelessness. *J Subst Abuse Treat*. 2022;138:108748. doi:10.1016/j.jsat.2022.108748

Shelley D, Cantrell J, Wong S, Warn D. Smoking cessation among sheltered homeless: a pilot. *Am J Health Behav*. 2010;34(5):544-552. doi:10.5993/ajhb.34.5.4

Sifat MS, Alexander AC, Businelle MS, et al. E-Cigarette switching and financial incentives to promote combustible cigarette cessation among adults accessing shelter services: A pilot study. *Drug Alcohol Dep Rep.* 2024;13:100295. doi:10.1016/j.dadr.2024.100295

Skelton E, Lum A, Cooper LE, et al. Addressing smoking in sheltered homelessness with intensive smoking treatment (ASSIST project): A pilot feasibility study of varenicline, combination nicotine replacement therapy and motivational interviewing. *Addict Behav*. 2022;124:107074. doi:10.1016/j.addbeh.2021.107074

Sung H, Apollonio DE. Evaluation of Tobacco Control Policies in San Francisco Homeless Housing Programs. *Health Promot Pract*. 2017;18(4):571-580. doi:10.1177/1524839917705128

Tucker J, Linnemayr S, Pedersen E, et al. Pilot Randomized Clinical Trial of a Text Messaging-Based Intervention for Smoking Cessation Among Young People Experiencing Homelessness. *Nicotine Tob Res*. 2021;23(10):1691-1698. doi:10.1093/ntr/ntab055

Vijayaraghavan M, Apollonio DE. Engaging Adults Experiencing Homelessness in Smoking Cessation Through Large-Scale Community Service Events. *Health Promot Pract*. 2019;20(3):325-327. doi:10.1177/1524839919835280

Vijayaraghavan M, Guydish J, Pierce J. Building Tobacco Cessation Capacity in Homeless Shelters: A Pilot Study. *J Community Health*. 2016;41(5):998-1005. doi:10.1007/s10900-016-0182-x

Vijayaraghavan M, Hurst S, Pierce JP. A Qualitative Examination of Smoke-Free Policies and Electronic Cigarettes Among Sheltered Homeless Adults. *Am J Health Promot*. 2017;31(3):243-250. doi:10.4278/ajhp.150318-QUAL-781

Vijayaraghavan M, Pierce JP. Interest in Smoking Cessation Related to a Smoke-Free Policy Among Homeless Adults. *J Community Health*. 2015;40(4):686-691. doi:10.1007/s10900-014-9985-9

Wilson SM, Blalock DV, Young JR, et al. Mobile health contingency management for smoking cessation among veterans experiencing homelessness: A comparative effectiveness trial. *Prev Med Rep*. 2023;35:102311. doi:10.1016/j.pmedr.2023.102311
